# RBP4: A Culprit for Insulin Resistance in End Stage Renal Disease That Can Be Cleared by Hemodiafiltration

**DOI:** 10.1155/2017/7270595

**Published:** 2017-11-23

**Authors:** Fabrizio Grosjean, Pasquale Esposito, Rosario Maccarrone, Carmelo Libetta, Antonio Dal Canton, Teresa Rampino

**Affiliations:** Nephrology, Dialysis, Transplant, Fondazione IRCCS Policlinico San Matteo and University of Pavia, Pavia, Italy

## Abstract

**Introduction:**

Retinol Binding Protein 4 (RBP4) is mainly excreted by the kidney and plays a pivotal role in insulin resistance (IR). In our study, we evaluated the association between RBP4 and IR in hemodialysis subjects (HD). We also assessed how circulating RBP4 could be influenced by kidney transplant or different dialytic techniques.

**Methods:**

RBP4 serum levels were evaluated in HD (*n* = 16) and matched healthy controls (C; *n* = 16). RBP4 and glucose transporter type 4 (GLUT4) mRNA expressions were also determined in adipose tissue. Circulating RBP4 was evaluated after kidney transplant (*n* = 7) and in hemodialysis patients (*n* = 10) enrolled in a cross-over study treated with standard bicarbonate dialysis (BD) or hemodiafiltration (HDF).

**Results:**

HOMA index (*P* < 0.05) and serum RBP4 (*P* < 0.005) were higher in HD compared to C. RBP4 levels positively correlated with fasting serum glucose (*P* < 0.05). RBP4 mRNA was lower in HD compared to C (*P* < 0.05) and positively correlated with kidney function (*P* < 0.05) and GLUT4 mRNA (*P* < 0.001). Transplant or HDF reduced circulating RBP4 (*P* < 0.01 and *P* < 0.05, resp.). Our results demonstrate that IR is associated with high circulating RBP4 and that suppressed RBP4 adipose tissue expression is accompanied by reduced GLUT4 expression in HD. Renal transplantation or HDF are effective in lowering serum RBP4 levels.

## 1. Introduction

Insulin resistance (IR) is common in patients with chronic kidney disease (CKD) and it often occurs in early stages of the renal impairment (glomerular filtration rate < 60 mL/min) [1]. IR is defined by normal fasting serum glucose associated with high serum insulin levels, a state that results in type 2 diabetes when the increased insulin secretion is no longer able to compensate for impaired peripheral insulin responsiveness [2]. IR has a particularly relevant clinical impact in End Stage Renal Disease (ESRD) because it adds independent cardiovascular risk in patients who are rated as having an inherent increased cardiovascular (CV) risk and 10-time higher mortality for CV events than the general population [3–6]. As yet, the mechanisms underlying IR in CKD are far from being fully understood. Possible culprits include low tolerance to physical activity, sedentary life style, toxins no longer cleared by the failing kidneys, and an endocrine dysfunction of adipose tissue [7–12]. Retinol Binding Protein 4 (RBP4) is a 21 KDa protein produced by the adipocytes that is secreted in the circulation where it binds transthyretin, giving origin to 80 KDa complex that carries vitamin A (retinol) to the target tissues. After having released retinol, this macromolecular complex splits up and RBP4 is freely filtered by the glomeruli; then it is partially reabsorbed and catabolized by the proximal tubules and it is excreted into the urine [13, 14]. Free RBP4 participates in glucose metabolism by inducing gluconeogenesis in liver cells and inhibiting glucose uptake in muscle cells. These combined effects raise plasma glucose and trigger insulin secretion, eventually resulting in the scenario of IR. Studies in rodents have shown that RBP4 production is inversely related to adipocyte expression of glucose transporter type 4 (GLUT4), an insulin dependent glucose transporter that induces glucose uptake in fat and muscle, and that GLUT4 expression is reduced in adipocytes but not in muscle cells in IR [8, 12, 15–17]. It has also been shown that RBP4 can reduce GLUT4 expression in adipocytes and that it can indirectly inhibit insulin signalling in adipocytes by inducing release of inflammatory cytokines in macrophages [18, 19]. Furthermore, high RBP4 levels have been observed in obese subjects, type 2 diabetic patients, adolescents with cardiovascular risk factors, and nonobese individuals low insulin sensitivity suggesting a pathogenic link between RBP4 and IR in humans [20–23]. Based on these findings we have investigated the association between RBP4 and IR in ESRD patients. Because RBP4 is mainly excreted by the kidney and it has been described that molecules with molecular weight similar to RBP4 are cleared efficiently by hemodiafiltration (HDF) rather than by standard bicarbonate dialysis (BD), we assessed how RBP4 levels are influenced by kidney transplant or different extracorporeal dialysis techniques.

## 2. Materials and Methods

### 2.1. Study Design

We enrolled patients undergoing standard hemodialysis (HD) for at least 3 months in our Unit of Nephrology, who were on kidney transplant waiting list. Exclusion criteria were diabetes or glucose intolerance, malignancies, acute and chronic active infections, malnutrition, and failure of the vascular access. Matched healthy subjects were enrolled as controls (C). Normal renal function was defined as normal serum creatinine (lower than 1.2 mg/dL) and glomerular filtration rate (GFR) estimated with Cockroft and Gault formula greater than 60 mL/min with normal urinalysis. Malnutrition, diabetes, and impaired glucose tolerance were defined according to established criteria [24–26]. To evaluate the effect of different dialysis technique on RBP4 circulating levels we included in the present study further unselected ESRD patients enrolled in a previous cross-over study (BD versus HDF, 3-month treatment, *n* = 10). Age, sex, 24 h diuresis for hemodialysis (HD), history of hypertension, cardiovascular events, smoking, alcohol abuse, physical activity and regular drug use, malignancies and active infections, familiar history of diabetes, and cardiovascular events were recorded at the enrolment. Alcohol abuse was defined as consumption greater than 25 g per day, residual diuresis in HD patients as a urine volume greater than 500 mL per day, and physical activity as aerobic exercises for at least 30 min three times a week. We measured blood pressure, heart rate, body weight, and height and we derived the Body Mass Index (BMI). The degree of insulin resistance/sensitivity was derived with the “homeostatic model assessment (HOMA) index”: fasting serum glucose [mMol/L] × fasting serum insulin/22.5 [26]. The protocol was approved by the Ethical Board of our Institution (P-20080008696), and it was funded by the Fondazione IRCCS Policlinico San Matteo (08054303/10).

### 2.2. Serum RBP4 Levels Quantification

Blood samples were obtained from all HD patients soon before the dialysis session and at the end of the same session when needed to evaluate dialysis RBP4 removal. Blood samples were also obtained from HD patients receiving kidney graft 12 months after transplant. RBP4 was measured in serum obtained from 3 ml of blood and then stored at –80°C within 30 min of sampling. Serum RBP4 levels were quantified by ELISA (Pantec, Marburg, Germany) following manufacturer instructions.

### 2.3. Adipose RBP4 and GLUT4 Gene Expression

RBP4 and GLUT4 mRNA expression was evaluated in adipose tissue obtained from 13 subjects, 7 were on HD and 6 were healthy controls (C). 4 HD subjects were matched with 4 controls for age, sex, and BMI. Adipose tissue was collected from HD patients during kidney transplant surgical procedure soon before the graft, while control adipose tissue was obtained from healthy subject during cold hernia surgical correction. Fat samples were snap frozen and stored at −80°C. RNA was extracted from adipose tissue using RNeasy Lipid Tissue Mini Kit (Qiagen, Venlo, The Netherlands), quantified, and retrotranscribed to cDNA (First Strand cDNA Synthesis Kit for RT-PCR, Roche, Basel, Switzerland). Quantitative polymerase reaction (qRT-PCR) was performed using the following primers: RBP4 (Hs00198830_m1) and GLUT4 (Hs00168966_m1, Applied Biosystems, Carlsbad, California, USA). GAPDH was employed as housekeeping gene. Gene expression was quantified by ΔΔCt methods as previously described [27, 28].

### 2.4. Statistical Analysis

From the available data we hypothesized mean serum RBP4 levels in HD twice greater than in C [22, 23, 29–31] and a standard deviation equal to the half of the mean; thus we estimated to enrol at least 15 participants per group in order to achieve a power greater than 90% in stating a difference between the two groups. We could not find any information on tissue expression of RBP4 and GLUT4 useful to build up a statistical prediction. The Shapiro-Wilk's test was used to test the normal distribution of quantitative variables. If they were normally distributed, mean and standard deviation (SD) were used to summarize the results; otherwise, we used median and interquartile range (IQR; 25°–75° percentile). Parametric or nonparametric tests were used to compare quantitative variables (*t*-test or Man-Whitney* U* test for independent data for comparisons between two groups and Anova or Kruskall Wallis test for comparisons among more than two groups). The chi-squared statistics or Fisher's exact test, as appropriate, were applied to compare qualitative variables. *P* < 0.05 was considered statistically significant. Pearson's *r* coefficient was used to evaluate the correlation between parameters. All tests were two-sided. Data analysis was performed with STATA statistical package (version 9; Stata Corporation, College Station, 2008, Texas, USA).

## 3. Results

### 3.1. Demography

We enrolled 16 regular HD patients (51.5 (44.0–59.7) years, 12 males) with the mean duration of dialysis being 28 ± 22 months, of whom 7 underwent renal transplantation after their enrolment. 16 healthy subjects (51.0 (44.0–58.2) years, 12 males) were the C. As shown in Table 1 the baseline demographic characteristics of the two groups were similar: 75% were male with no differences regarding age and BMI, personal and familiar history of cardiovascular events, diabetes, lipid disorders, smoking, and alcohol ingestion. There were no differences in total serum protein levels, serum albumin, and total serum cholesterol, whereas serum triglyceride levels were significantly higher in HD group (*P* < 0.05). Not surprisingly history of hypertension, systolic blood pressure, and physical inactivity were more prevalent in HD compared to C (*P* < 0.005, *P* < 0.05, and *P* < 0.005, resp.), furthermore serum hemoglobin levels were significantly lower while serum iron levels where higher in HD compared to C (*P* < 0.005 and *P* < 0.05), respectively. Among HD, 94% of the patients had arterial-venous fistula as vascular access and 53% had residual diuresis of more than 500 ml/24 hours. Of note C reactive protein (CRP) was normal in all the HD subjects (0.5 ± 0.4 mg/dL; n.v. <0.8 mg/dL).

### 3.2. Insulin Resistance Trait Characterizes ESRD Patient on Hemodialysis

**Figure 1:**
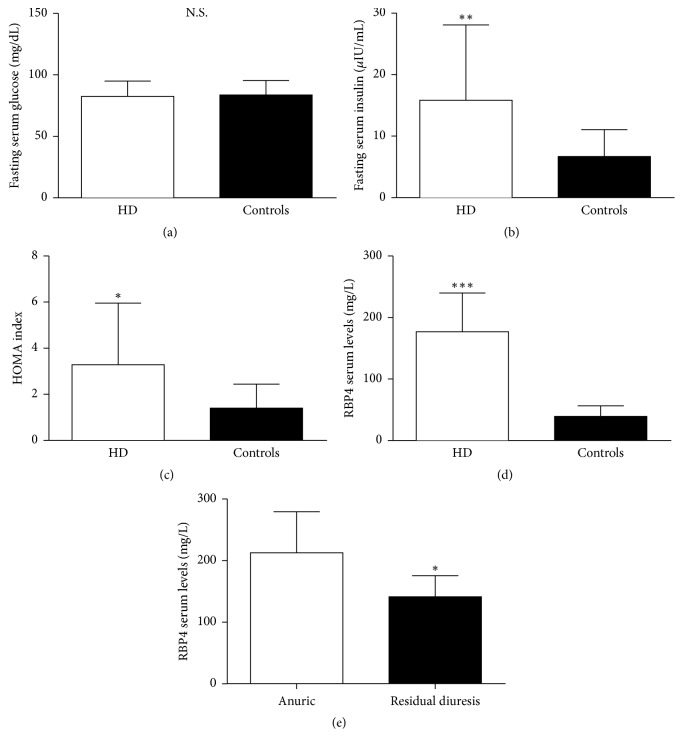
Fasting serum glucose levels (a), fasting serum insulin levels (b), HOMA index (c), and serum RBP4 (d) in hemodialysis patients (HD) and controls. Influence of residual diuresis on serum RBP4 in HD patients (e). ^*∗*^*P* < 0.05, ^*∗∗*^*P* < 0.01, and ^*∗∗∗*^*P* < 0.005.

All HD patients and all the controls (C), but one, had normal glycosylate hemoglobin (<5.9%, according to our laboratory limits); all the participants had normal fasting serum glucose levels (<110 mg/dL) (Table 1) with no differences between the two groups (82.6 ± 12.3 mg/dL in HD versus 83.8 ± 11.8 mg/dL in C, NS; Figure 1(a)). On the contrary, fasting serum insulin levels and HOMA index were significantly higher in HD compared to C (15.8 ± 12.3 versus 6.7 ± 4.4 *μ*IU/ml, *P* < 0.01; 3.3 ± 2.7 versus 1.4 ± 1.0, *P* < 0.05, resp.; Figures 1(b) and 1(c)).

### 3.3. RBP4 Serum Levels Are Elevated in ESRD Patient on Hemodialysis

**Figure 2:**
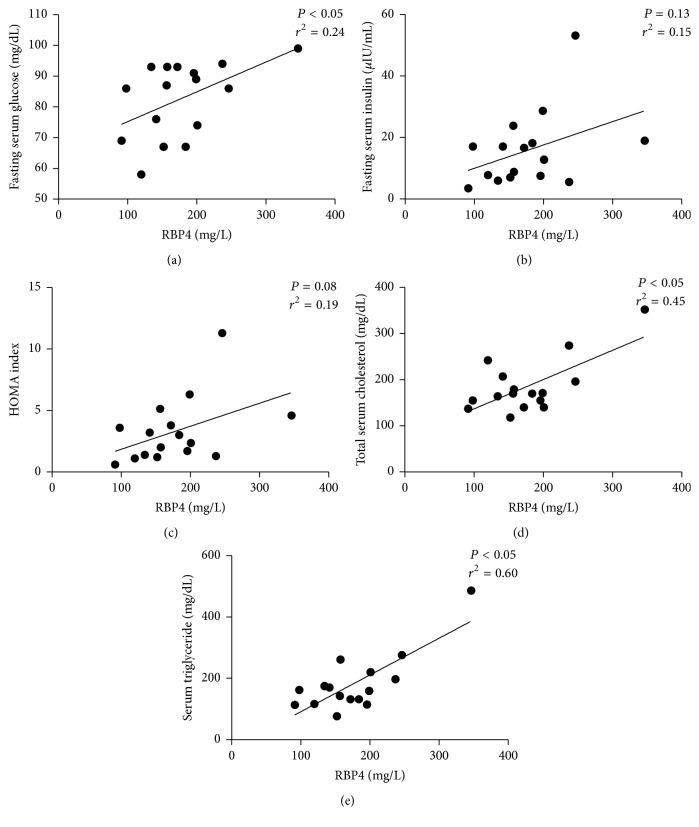
Correlation between serum RBP4 levels and fasting serum glucose (a), fasting serum insulin (b), HOMA index (c), total serum cholesterol (d), and serum triglycerides (e) in hemodialysis patients (HD).

Serum RBP4 levels were four times higher in HD compared to C (176.9 ± 63.2 versus 39.2 ± 17.4 mg/L, *P* < 0.005; Figure 1(d)); furthermore, serum RBP4 levels were lower in HD with preserved residual diuresis compared to the anuric HD patients (141.3 ± 34.2 versus 212.5 ± 67.0 mg/L; *P* < 0.005; Figure 1(e)). Serum RBP4 levels correlated significantly and directly with fasting serum glucose levels (*P* < 0.05, *r*^2^ = 0.24; Figure 2(a)) but not with insulin levels and HOMA index (*P* = 0.13 and *P* = 0.08, resp.; Figures 2(b) and 2(c)) in HD group. Furthermore, serum RBP4 levels correlate directly with total serum cholesterol (*P* < 0.05, *r*^2^ = 0.45; Figure 2(d)), serum triglycerides (*P* < 0.05, *r*^2^ = 0.60; Figure 2(e)), and heart rate (*P* = 0.05, *r*^2^ = 0.26).

### 3.4. Adipose Tissue RBP4 and GLUT4 mRNA Expression Are Decreased in ESRD Patient

**Figure 3:**
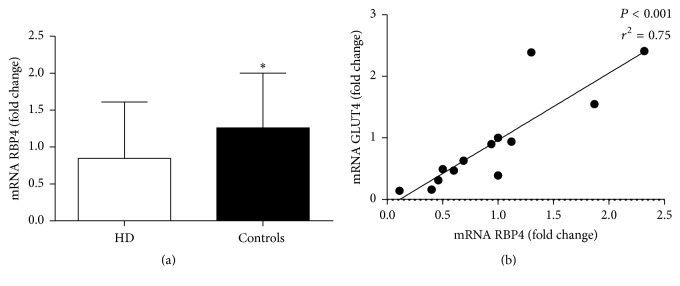
Adipose tissue RBP4 mRNA expression (a) in hemodialysis (HD) and controls. Correlation between adipose tissue RBP4 and GLUT4 mRNA expression. ^*∗*^*P* < 0.05.

Subcutaneous adipose RBP4 mRNA became lower in HD compared to matched sex, age, and BMI controls (*P* < 0.05, Figure 3(a)), while GLUT4 mRNA did not differ between HD and C (0.56 ± 0.40 versus 1.05 ± 0.94 fold change, *P* = 0.25). RBP4 mRNA correlated positively with subcutaneous GLUT4 expression (*P* < 0.001 and *r*^2^ = 0.75, Figure 3(b)) and negatively with age (*P* < 0.01 and *r*^2^ = 0.47) in the overall population. RBP4 mRNA correlated positively with kidney function (eGFR, *P* < 0.05  *r*^2^ = 0.72) in controls. Moreover GLUT4 expression showed negative correlations with age and BMI (*P* < 0.05 and *r*^2^ = 0.31, *P* < 0.01 and *r*^2^ = 0.47, resp.) in the overall population.

### 3.5. RBP4 Serum Levels Are Reduced after Kidney Transplant

**Figure 4:**
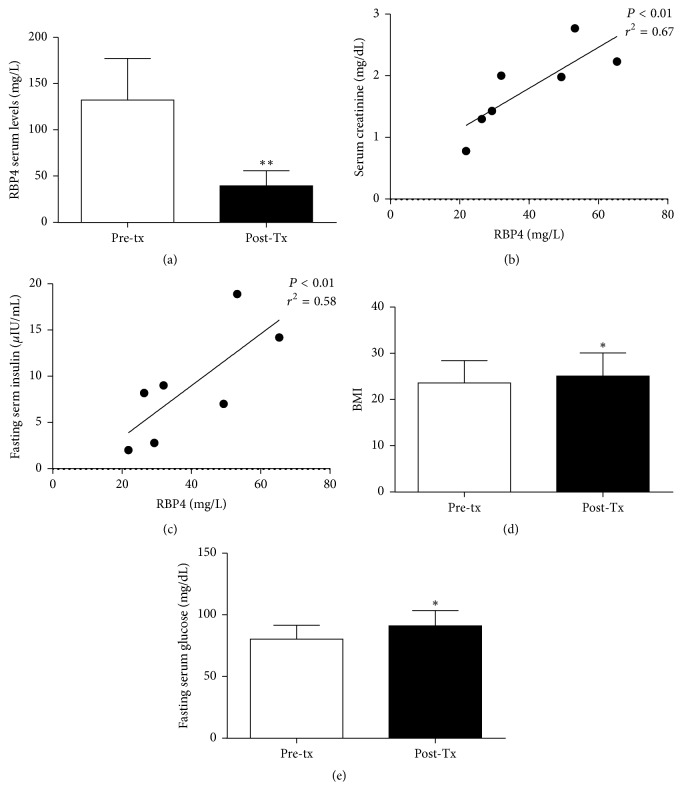
RBP4 serum levels (a) before (Pre-TX) and after kidney transplant (Post-TX). Correlation between serum RBP4 levels and serum creatinine (b); fasting serum glucose levels (c) in kidney transplanted patients. Body Mass Index (BMI) (d) and fasting serum glucose levels (e) before (Pre-TX) and after kidney transplant (Post-TX). ^*∗*^*P* < 0.05, ^*∗∗*^*P* < 0.01.

In the 7 patients undergoing renal transplantation serum RBP4 levels were significantly reduced compared to pretransplant levels (39.6 ± 16.3 versus 132.2 ± 45.2 mg/L, resp., *P* < 0.01; Figure 4(a)); these levels positively correlated with serum creatinine and fasting serum insulin (*P* < 0.01 and *r*^2^ = 0.67; *P* < 0.01 and *r*^2^ = 0.58, resp.; Figures 4(b) and 4(c)). All the patients showed normal fasting serum glucose (90 ± 11 mg/dL) after kidney transplant and serum creatinine was 1.5 ± 0.64 mg/dL. Despite the significant increase of the BMI (23,6 ± 4.8 versus 25.1 ± 5 kg/m^2^, *P* < 0.05; Figure 4(d)) accompanied by the increase of fasting serum glucose serum levels (80.3 ± 11.5 versus 91.1 ± 12.4 mg/dL; Figure 4(e)), serum fasting insulin, HOMA index, total serum cholesterol, and serum triglyceride did not change significantly (12.6 ± 8.2 versus 8.9 ± 6.0 *μ*IU/ml, 2.5 ± 1.8 versus 2.0 ± 1.5, 187.9 ± 55.7 versus 182.4 ± 52.9 mg/dL, and 171.1 ± 46.4 versus 99.4 ± 27.2 mg/dL, resp.; NS).

### 3.6. HDF Lowers RBP4 Circulating Levels in ESRD Patients

**Figure 5:**
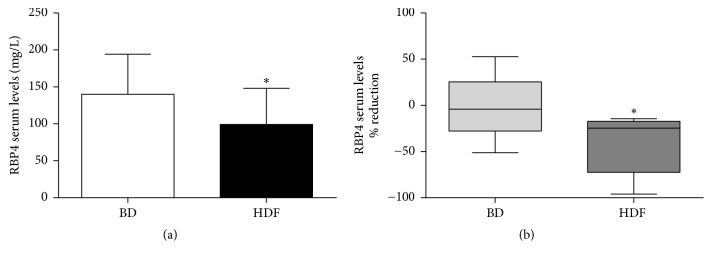
Effect of HDF treatment on RBP4 circulating levels (cross-over study, a). RBP4 intradialysis removal (b). ^*∗*^*P* < 0.05.

In the patients enrolled in the cross-over study RBP4 circulating levels were significantly reduced by HDF treatment compared to BD (99.2 ± 48.9 versus 140.1 ± 54.1 mg/L, *P* < 0.05; Figure 5(a)). Furthermore, HDF treatment granted 20-fold greater intradialysis RBP4 removal compared to BD (Figure 5(b)).

## 4. Discussion

**Figure 6:**
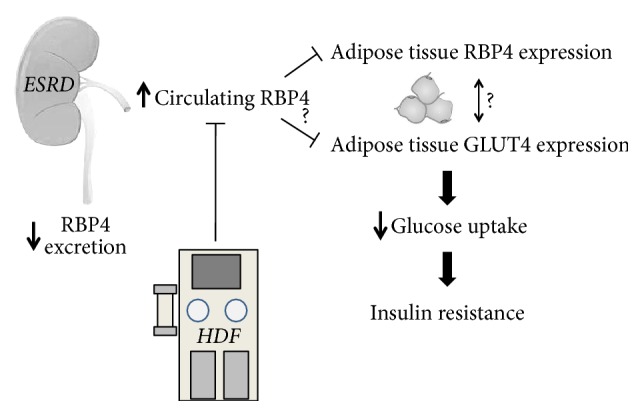
Hypothesis about pivotal role of the kidney in RBP4 mediated insulin resistance (IR) in End Stage Renal Disease (ESRD): possible intervention by HDF.

Our study shows that serum RBP4 levels are four times higher in ESRD patients on maintenance hemodialysis compared to subjects with normal renal function. The kidney is the main site of RBP4 clearance because RBP4 is filtered by the glomeruli and it is further metabolized by tubular cells. Therefore, it can be hypothesized that defective RBP4 renal clearance is the main cause of RBP4 accumulation in ESRD patients and that RBP4 retention is not obviated by standard bicarbonate dialysis because its molecular weight is in the range of middle molecules [13]. As expected from studies on renal handling of middle molecules, residual diuresis preserved some RBP4 excretion and was associated with lower serum levels of RBP4 [32]. Furthermore, an ultimate evidence of the strict dependence of RBP4 serum levels on renal function is provided by our findings that serum RBP4 levels decreased proportionally with renal function recovery in kidney graft recipients. Finally RBP4 gene was less expressed in adipocytes of HD compared to paired C, a result that strengthens the evidence that increased production of RBP4 in adipose tissue did not account for its high blood levels in HD. In our study we carefully excluded patients with any general risk factor for metabolic and cardiovascular disorders, but those associated with renal failure and dialysis. In particular to prevent sampling biases we matched patients and controls for sex, age, and BMI and we have enrolled only patients that were in the range advised by guidelines as for Hb levels, indexes of bone metabolism, and dialysis performance [25]. Normal blood glucose levels required a threefold increase of serum insulin levels in HD subjects compared to controls, a state of IR that was confirmed by the HOMA index. For the first time we show that serum levels of RBP4 correlated directly with fasting glucose in ESRD undergoing dialytic treatment. In the absence of alternative causes of IR, RBP4 remains the only identifiable culprit for IR in our patients and the retention of RBP4 assumes the role of main pathogenic mechanism for metabolic dysfunction in ESRD. Interestingly, RBP4 levels were significantly correlated also with serum cholesterol and serum triglyceride thus confirming its link with IR and metabolic syndrome [15, 17]. Finally we found a positive correlation between RBP4 and heart rate an indirect marker of adrenergic drive hyperactivation that invariably characterizes the different aspects of the metabolic syndrome [33]. It has been shown that RBP4 serum levels are associated with kidney function rather than type II diabetes in nondialytic population with moderate kidney disease [30]. However, our study provides some insights regarding RBP4 role on IR in a different setting characterized by severe kidney disease requiring hemodialysis. In this regard our data are confirmatory of previous findings in experimental studies in murine models of type II diabetes and uremia [15, 34]. High RBP4 serum levels and low adipose RBP4 mRNA in ESRD patients undergoing dialysis figure a feedback system where GLUT4 mRNA expression is also involved: excess RBP4 might operate a self-limitation by inhibiting RBP4 synthesis in one hand, while it inhibits GLUT4 expression contributing to insulin resistance in the other [35, 36]. These findings depict a scenario in which RBP4 and GLUT4 are reciprocally connected in a system that participates in glucose metabolism and whose disruption leads to RBP4-driven IR in the ESRD milieu [37]. The mechanisms that mediate the cross-talk between circulating RBP4 and its tissue expression, as well as the molecular transducers of RBP4, require full exploration in ESRD population. Our data show that poor RBP4 clearance can be attained with standard bicarbonate dialysis using dialyzers with low molecular cut-off; on the contrary we demonstrated that the dialyzer with higher cut-off employed in HDF can actually efficiently clear RBP4 (Figure 6). This observation is a sound base for future studies aimed to prove that RBP4 removal by HDF can modulate IR in ESRD patients.

## Figures and Tables

**Table 1:** Demographic characteristics.

	Hemodialysis	Controls	*P* value
Number of participants	16	16	—
Male, %	75%	75%	NS
Age, years (median, 25°–75° IQR)	51.5 (44.0–59.7)	51.0 (44.0–58.2)	NS
BMI, Kg/m^2^ (mean, ±SD)	24.4 ± 4.0	24.4 ± 3.0	NS
SBP, mmHg (mean, ±SD)	140 ± 26	122 ± 10	***0.02***
DBP, mmHg (mean, ±SD)	80 ± 13	78 ± 8	NS
Heart rate, bpm (mean, ±SD)	77 ± 11	70 ± 8	NS
History of cardiovascular events, %	14.6%	0	NS
Family history of cardiovascular events, %	60.0%	56.2%	NS
Family history of diabetes, %	40.0%	50.0%	NS
History of hypertension, %	81.25%	18.7%	***0.001***
History of lipid disorders, %	25.0%	18.7%	NS
Smoking, %	26.6%	12.5%	NS
Alcohol abuse, %	0%	6.2%	NS
Physical activity, %	13.3%	68.7%	***0.003***
Patients with normal fasting glucose	16/16	16/16	NS
Patients with normal glycated hemoglobin	14/14	14/15	NS
Total serum protein, g/dL (mean, ±SD)	6.9 ± 0.6	7.2 ± 0.2	NS
Albumin, g/dL (mean, ±SD)	4.3 ± 0.7	4.4 ± 0.1	NS
Hemoglobin, g/dL (mean, ±SD)	11.8 ± 1.5	14.1 ± 1.2	***0.001***
Serum iron levels, *μ*g/dL (mean, ±SD)	116.2 ± 47.8	85.6 ± 17.9	***0.02***
Total serum cholesterol, mg/dL (mean, ±SD)	185 ± 59	205 ± 34	NS
Serum triglyceride, mg/dL (mean, ±SD)	183 ± 97	109 ± 57	***0.01***

SBP = systolic blood pressure, DBP = diastolic blood pressure, and BMI = body mass index.
